# EBV and Its Associated Diseases: New Perspectives in the Post COVID-19 Era

**DOI:** 10.3390/v18040427

**Published:** 2026-04-01

**Authors:** Xiaoming Lyu

**Affiliations:** 1 Department of Laboratory Medicine, The Third Affiliated Hospital of Southern Medical University, Guangzhou 510630, China; xiaomlyu@smu.edu.cn; 2 The Third School of Clinical Medicine, Southern Medical University, Guangzhou 510630, China

The unprecedented global intersection of the COVID-19 pandemic and the persistent burden of Epstein–Barr virus (EBV) has created a unique inflection point in viral research. This Special Issue, titled “EBV and Its Associated Diseases: New Perspectives in the Post-COVID-19 Era,” was conceived to explore how the landscape of EBV-associated pathologies—from infectious mononucleosis to complex malignancies and autoimmune disorders—has been reshaped by the immunological shifts in the last several years.

The nine contributions gathered here represent a diverse cross-section of current research, spanning epidemiological shifts, molecular mechanisms, clinical rarities, and the next generation of therapeutic interventions.

## 1. The Post-Pandemic Epidemiological Landscape

One of the most immediate questions addressed in this issue is how pandemic-era public health measures altered the transmission and clinical presentation of EBV. Amarillo et al. provide critical insights into how social distancing and hygiene measures shifted EBV epidemiology, potentially delaying primary infection and altering the age demographics of those affected [1]. On a molecular level, the interaction between SARS-CoV-2 and EBV is explored by Mahajan et al. [2]. and Alsaadawe et al., who review how the “dual threat” of these viruses may contribute to immune dysregulation, Long COVID symptoms, and potentially heightened oncogenic risks [3].

## 2. Molecular Insights into Pathogenesis and Reactivation

A significant portion of this issue delves into the subtle regulatory mechanisms that allow EBV to transition from latency to lytic reactivation—a process increasingly scrutinized in the context of post-viral syndromes. Li et al. identify Hsa-miR-7974 as a crucial suppressor of EBV reactivation by directly targeting the immediate-early genes BZLF1 and BRLF1 [4]. In the context of EBV-associated gastric cancer, Dirimtekin et al. map the profiles of viral BART microRNAs, providing a clearer picture of how these viral non-coding RNAs manipulate host gene expression to favor tumor progression [5].

## 3. Clinical Manifestations and Rare Complications

EBV remains a “cellular hijacker” capable of diverse clinical outcomes [6]. Two case reports in this issue highlight the virus’s unpredictable nature: Jugulete et al. describe rare cases of reversible hypoglossal nerve palsy following infectious mononucleosis, underscoring the neurological complexities of acute infection [7]. Carpani et al. report a unique instance of amoxicillin-induced atypical exanthema in a patient with EBV-related nasopharyngeal carcinoma, illustrating the continued relevance of classic clinical dilemmas in specialized oncology settings [8].

## 4. Advancing Diagnostics and Therapeutics

Finally, this Special Issue looks toward the future of clinical management. Sun et al. offer a comprehensive review of the evolution of EBV detection, transitioning from traditional serology to modern, highly sensitive molecular technologies [9]. In a leap toward precision therapy, Lin et al. demonstrate the potential of CRISPR/Cas13-mediated inhibition of EBNA1 to suppress the viral load and transcripts in nasopharyngeal carcinoma cells, offering a promising alternative to conventional chemo-radiotherapy [10].

## 5. Conclusions

The research presented in this Special Issue confirms that the post-COVID-19 era is not merely a return to “business as usual” for EBV research. Instead, it is a period of renewed urgency. The interplay between viral reactivation and systemic inflammation, the shifting ages of primary infection, and the advent of CRISPR-based therapies all suggest that our approach to EBV-associated diseases must be as adaptive as the virus itself.

## Data Availability

This is an editorial article. No new data were created or analyzed. All information discussed is available within the article or from the cited references.
